# [Corrigendum] Endogenous Nampt upregulation is associated with diabetic nephropathy inflammatory-fibrosis through the NF-κB p65 and Sirt1 pathway; NMN alleviates diabetic nephropathy inflammatory-fibrosis by inhibiting endogenous Nampt

**DOI:** 10.3892/etm.2026.13238

**Published:** 2026-07-08

**Authors:** Ye Chen, Yuzhen Liang, Tingting Hu, Riming Wei, Congjie Cai, Ping Wang, Lingyu Wang, Wei Qiao, Leping Feng

Exp Ther Med 14:4181–4193, 2017; DOI: 10.3892/etm.2017.5098

Following the publication of this paper, it was drawn to the Editor’s attention by a concerned reader that the Sirt1 western blot data shown in Fig. 4A on p. 4187 were strikingly similar to the Sirt1 western blot data shown in Fig. 6A on p. 4188, even though the experimental conditions reported for these figure parts were apparently different.

Upon examining the original data in their paper, the authors have realized that the Sirt1 data correctly featured in Fig. 4A had inadvertently been re-used in Fig. 6A. The authors have repeated the western blots for Sirt1 protein in Fig. 6A, and the results obtained were broadly similar to those obtained originally. The revised version of Fig. 6, featuring the new data for Sirt1 in Fig. 6A, is shown below. All the authors agree to the publication of this Corrigendum, and are grateful to the Editor of *Experimental and Therapeutic Medicine* for granting them the opportunity to publish this; moreover, they apologize to the readership for any inconvenience caused.

## Figures and Tables

**Figure 6:**
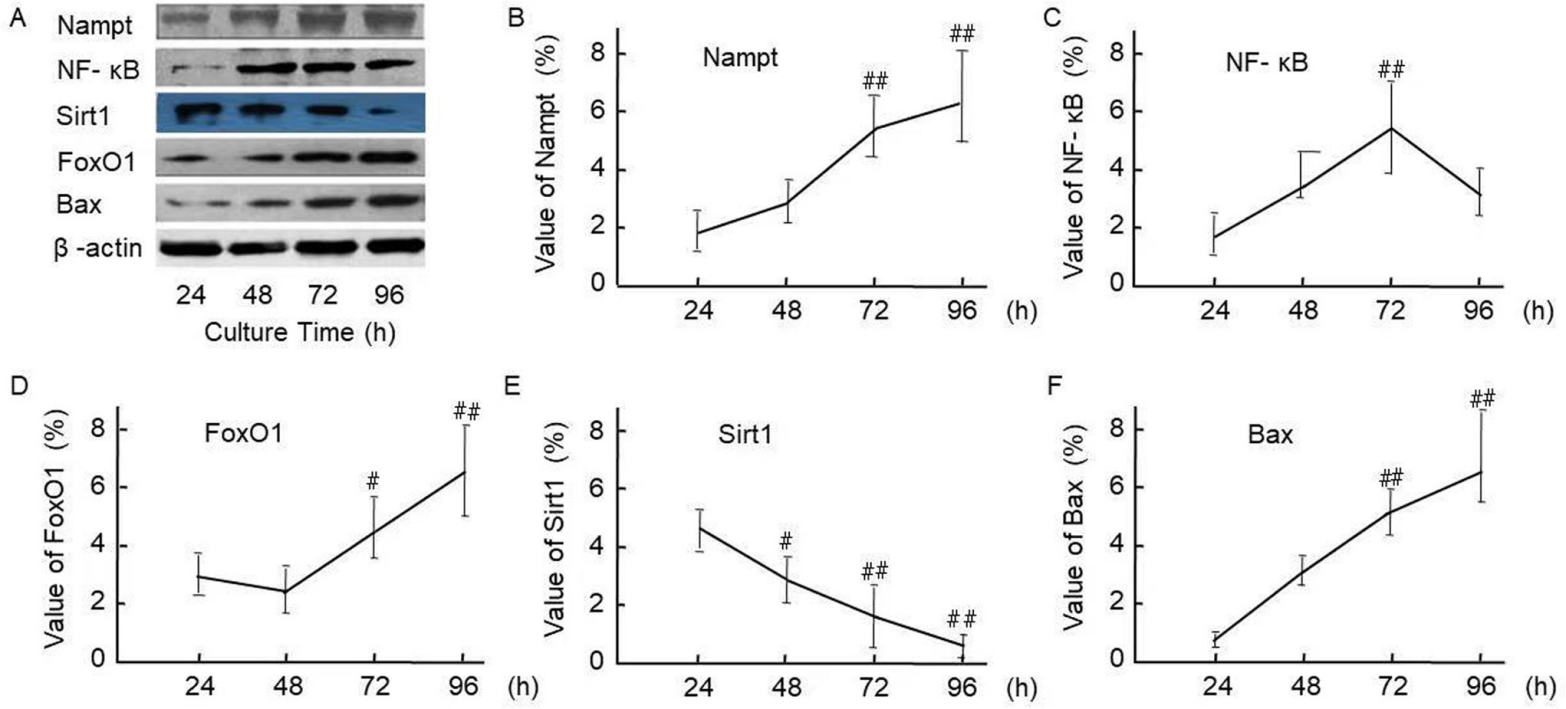
(A) Time course of endogenous Nampt, NF-κB p65, Sirt1, FoxO1, and Bax protein expression in HBZY-1 cells exposed to a high-glucose milieu for 24, 48, 72, or 96 h. Quantification of western blot analysis indicated the protein expression levels of (B) Nampt, (C) NF-κB p65, (D) Sirt1, (E) FoxO1 and (F) Bax, and β-actin were indicated. Data are presented as means ± standard deviations of three experiments. ^#^P<0.05, ^##^P<0.01 vs. 24 h (control). Nampt, nicotinamide phosphoribosyltransferase; NF, nuclear factor; Sirt1, sirtuin 1; FoxO1, forkhead box protein O1; Bax, B-cell lymphoma 2-like protein 4.

